# Debates in allergy medicine: Specific immunotherapy in children with atopic dermatitis, the “con” view

**DOI:** 10.1186/s40413-016-0107-2

**Published:** 2016-04-18

**Authors:** David N. Ginsberg, Lawrence F. Eichenfield

**Affiliations:** Department of Dermatology, Rady Children’s Hospital, San Diego, CA USA; Department of Dermatology, University of California, San Diego, La Jolla USA; Division of Dermatology, Rady Children’s Hospital, San Diego, CA USA

**Keywords:** Specific immunotherapy, Pediatric, Atopic dermatitis, Food allergy, Environmental allergy

## Abstract

Atopic dermatitis (AD) is a common chronic skin condition in children that has a proven association with other atopic conditions and allergies. These associations, like the general pathophysiology of AD, are complex and not fully understood. While there is evidence for the efficacy of specific immunotherapy (SIT) in pediatric asthma and allergic rhinitis (AR), there is a lack of strong data to support its use in AD. IgE has been shown to be elevated in many patients with AD, but it is an unreliable biomarker due to variability and great fluctuation over time, poor positive predictive value for clinically relevant allergy, and poor correlation with disease state. In spite of this, almost all studies of SIT use either positive skin prick testing (SPT) or serum specific IgE levels to guide therapy. Allergen avoidance, with some exceptions, is generally not effective at controlling AD in children. The few studies that have investigated the efficacy of SIT in children with AD have produced conflicting results, and a lack of reproducibility with a standard treatment protocol. Limited studies have shown clinical improvement in mild to moderate AD cases, but no effect on more severe patients. Uncontrolled studies are difficult to interpret, due to the natural history of remission or “outgrowing” of AD over time in many patients without specific interventions. Drawbacks to SIT include the length of treatment, poor compliance, cost, and potential side effect profile. The potential for misdirection of time and energy away from skin directed therapy could negatively impact on AD outcomes.

## Background

Atopic dermatitis (AD) is one of the most common chronic inflammatory skin conditions of children. The pathophysiology is complex and multifactorial, and is still not fully understood. There is work showing that epithelial barrier dysfunction, such as filaggrin deficiencies, may be genetically mediated and are important risk factors for the development of AD and other atopic phenomena, including food allergy (FA) and asthma [1–3]. Increasing insight into the immunology of AD has expanded our understanding of the role of TH2 responses in AD, which may be strongly influenced by antigenic challenges [4]. Specific immunotherapy (SIT), directed at these immunologic responses, however, has not been shown to be a successful, long term treatment modality in children with AD, despite being clinically useful in allergic rhinitis (AR) and asthma [5].

### Problems with the assumption of casual association between sensitization to allergen and AD

Historically, many experts have divided AD into intrinsic and extrinsic. The concept of extrinsic AD is that some patients throughout the course of their AD are more affected by allergens, but interpretation of these labels is made uncertain by the data that shows that IgE sensitization may be present in many individuals without apparent clinical consequence [6]. While in other cases the IgE sensitization is associated with clinically significant allergies along a broad set of atopic manifestations, which includes urticarial, eczematous dermatitis, wheezing, proctocolitis, vomiting and AR [7].

For many patients AD is well known to be the first step of what is known as the atopic march, which includes AR and asthma [8]. Current literature shows that children with AD have an increased prevalence of atopy, or the tendency toward allergen induced IgE sensitivity, compared to the general pediatric population [9]. However, this association has proven to be quite complex and difficult to interpret, given the already complex pathophysiology of AD itself. A review of atopy in AD patients, focusing mostly on children, showed that the prevalence of atopy varied from 7–78 % across the literature, with a higher rate in hospitalized patients, who presumably had more severe diseases [9]. This large range makes it difficult to recommend the use of any treatment targeted specifically at the IgE immune response, when it is possible that so few patients within a given population would benefit from it. A further issue with many of the reviewed studies was the sole use of either specific serum IgE or skin prick testing (SPT) as the only confirmatory test for sensitization. This likely led to high false positive rates, or varying rates of positive tests without clinically relevant allergic responses. Studies have shown that, particularly in young children due to their developing immune system, each of these tests alone can be difficult to interpret and they can yield false results [10]. The positive predictive value (PPV) of SPT alone is less than 40 % when evaluating FA in AD patients and the specificity of SPT alone for environmental allergens is between 44–53 % [11, 12]. A recent review has also shown that there is a surprising lack of standardization of the practice of SPT by medical professionals, despite this technique having been used for over a century. This gives even more reason to question the validity of the research using SPT alone [13].

The currently held belief is that a diagnosis of sensitization with increased IgE response without any clinical manifestations is not clinically useful. Multiple studies have shown a trend of increasing total and specific IgE levels in more severe disease [9, 14]. However, one of the few studies done on the efficacy of immunotherapy in the pediatric AD population did show improvement but the only significant results were seen in the mild to moderate cases, with no clinical improvement seen in severe cases [15].

Although IgE may have an association with AD severity, using it as a marker for success of treatment with immunotherapy has not proven effective [16, 17]. IgE levels have been shown to fluctuate tremendously throughout childhood, especially in the first year of life, when they fluctuate due to environmental and maternal factors [18].

In one of the larger, multi-national, studies done specifically looking at sensitivities of infants with AD, Benedictis et al. showed that over half of the infants studied were sensitized to at least one common allergen [19]. They used serum specific IgE to test for sensitization; the data shows that less than 20 % of the infants with AD were monosensitized, or had a response to only one of the common allergens tested, and 36.8 % were polysensitized [19]. The practicality of a more generalized immunotherapy could be very different from the currently used SIT due to the low PPV of skin prick and serum IgE testing and the uncertainty of their positive results.

### Association between food allergy and AD

High rates of serum specific IgEs and positive SPT have been seen in AD patients and these levels appear to trend upward with increasing disease severity [16, 17]. Due to high rates of false positive testing, definitions of FA now require consistent clinical manifestations with food exposure to make a diagnosis, similarly, in individuals with AD, diagnosis of a FA should require consistent clinical response with food exposure, that may be eczematous dermatitis or other atopic manifestations. While numerous studies have documented eczema recurrence or flares during oral food challenges, the evidence is mixed about the impact of avoidance of FA on the course of AD [20–22]. This can be rather complex due to the uncertainty of possible significant late reactions, which are harder to observe and control in clinical circumstances.

Children with AD often outgrow their disease [23]. Thus it may be hard to know if improvement of AD in an individual is due to the natural disease progression or the effect of allergen avoidance. The objective evidence has not shown clinical utility of food restriction, other than egg, in children who had proven FA and AD [20].

An important study that may have a potential impact on the perspectives on FA and AD is the Learning Early About Peanut Allergy (LEAP) study that enrolled infants with either severe AD or egg allergy [24]. They performed SPT, enrolling patients with negative tests, or positive but less than 5 mm wheal size, into randomized groups. Within each of these groups, half of the patients were fed peanut products 3 times per week beginning early in life, and the other half had peanut products withheld entirely. In both of the groups that were fed peanuts, there was a marked reduction in the rates of clinical peanut allergy. Interestingly, in the LEAP study group there was no apparent impact or change in the AD time to resolution or eczema severity in the group that avoided peanuts, compared to those that had early exposure. Overall there was a poor correlation between specific IgE, skin prick testing, and the development of FA. This data does not support the avoidance of food in the context of patients who may be sensitized. While peanut allergy was prevented in many individuals, it did not show improvement of the eczema with this experimental allergen avoidance.

FAs in general have been shown to affect younger children more commonly, while environmental allergies are more common in adolescents and adults [25]. The reported prevalence of FAs among the AD population is about 15 %, which is three times higher than the rate among the general pediatric population [26, 27]. One of the few studies that assessed for FA in children with AD that was not hindered by selection bias, and included patients with a low suspicion of FA, found a prevalence of 30 % [28]. Close to 90 % of these FAs are to egg, milk, soy, nuts, or wheat.

Both FA and AD have been shown to improve spontaneously throughout childhood. Hence, any type of SIT based on FA would be controversial because it is difficult to predict the potential progression of the disease at a young age, and given the extensive length of treatment required for SIT, with the most commonly accepted duration being 1–3 years, it might be more prudent to observe and treat with more conservative management in early childhood [29].

### Previous studies on the clinical efficacy of allergen-specific immunotherapy for children with AD

Immunotherapy as a treatment for FA is currently under investigation. The results, though promising, have been shown through meta-analysis to be insufficient to fully assess the efficacy of this treatment as acceptable [30]. There have not been studies assessing AD as a sole allergic disorder, as oral immunotherapy studies have been carried out in both children and adults with multiple symptoms including anaphylaxis, asthma, generalized urticaria, and AD. There has also not been an investigation into whether immunotherapy directed at FA in children has any effect on their AD. The current expert consensus for food allergen testing in children with AD, is to test only if there is reason for suspicion [31]. This could include AD that persists in spite of optimized management and topical therapy, or a history of immediate reaction after ingestion of a specific food [31]. This same expert panel does not recommend immunotherapy as a treatment for FA.

Environmental allergens are thought by many to play an even larger role in AD pathophysiology than food allergens. The current research linking filaggrin mutations and the associated epithelial defects with AD are helping to support this belief [3]. This may have led to a greater focus on the research of SIT directed at environmental allergens for children with AD. There is also convincing evidence that clearly link controlled aeroallergen exposure to AD exacerbations [32]. This particular study was done in adult patients with grass pollen allergies. But the conditions were controlled well enough within a challenge chamber that we believe that the exacerbations were genuine and that the results can be extrapolated to pediatric AD patients as well. The relative significance of exacerbating factors for AD is known to differ depending on the patient’s age, and exposure to aeroallergens, including house dust mites, pets, and pollen, increases the severity of AD in school age children [33].

Of the environmental allergens that AD patients are sensitized to, house dust mites (HDM) are widely accepted as the most common in AD patients [29]. The majority of clinical trials looking into SIT as a potential treatment for AD focus solely on HDM SIT [29, 34]. There are currently very few studies that have looked into the efficacy of SIT as a treatment modality for children with AD, and there is conflicting data among them. Arguably, the best designed of these studies was a randomized, double blind, placebo controlled trial conducted by Pajno et al. Their inclusion criteria did allow for the patients to be sensitized to pollen or food allergens, but they ruled out any patient with a clinically significant allergy to anything other than HDM [15]. After their 18-month trial period, there was significant improvement in both the standardized clinical severity scoring system for AD (SCORAD) and in the use of rescue medication in the active sublingual immunotherapy (SLIT) group when compared to the placebo control. However, upon further analysis, when the subjects were divided between mild/moderate AD and severe AD, a statistically significant difference was only seen in the mild/moderate group and not in the severe group, compared to the placebos. Atopy and IgE sensitization appear to play a larger role in severe AD cases [17]. The inability to show efficacy of SIT for children with severe AD is a major detractor from its possible use as a treatment modality moving forward. Two of the other pediatric HDM SIT studies, one focusing on SLIT [35] and the other on Subcutaneous SIT (SCIT) [36] showed no significant clinical benefit. In one of these trials a second study was done using the initial treatment group, which did show significant improvement after the first 8-month trial. In this follow up study, however, the n was reduced, making the study underpowered and the trial became unblinded in order to continue studying the treatment group [36]. Both studies that showed no statistical difference between SIT and control also showed surprising improvement in the control groups, one being a placebo and the other just standard AD treatment [35, 36]. One final study that had an entirely pediatric population, that is often cited, was actually investigating the effects of HDM SIT on children with asthma, however there were subjects with AD, and although there was a subjective improvement in AD symptoms, it was not statistically significant [37].

More SIT studies focused on pediatric AD are needed to either prove reproducibility of positive results or to definitively deny its efficacy. Although HDM has garnered the most attention for pediatric AD SIT and shows the most promise in the adult AD population, without an investigation into other common environmental and food allergens, it is difficult to recommend SIT as a treatment option for children with AD [34]. Other limitations to the current studies include a lack of a standardized regimen of treatment leading to heterogeneity between the trials making any kind of meta-analysis of these smaller pediatric trails impossible.

### Future perspectives for studies on the specific immunotherapy for children with AD

Without an established biomarker to attempt to predict the outcome of treatment before it has begun, it will remain difficult to know which patients within the pediatric population could benefit from SIT. Currently only pediatric AD patients with IgE hypersensitivity, confirmed by questionable tests, have been investigated without producing definitive results. All of the current literature is relying on IgE and has not produced encouraging results, but the answer may lie elsewhere. One study of SIT in pollen allergies shows that a ratio of IgG4 to IgG1 can be used to predict outcomes better than IgE [38]. While another randomized controlled trial of SIT in AD patients, though not limited to children, showed no change in IgE, but saw a significant increase in IgG4 levels [39]. Attempting to focus so specifically on one potential allergic exacerbation of AD such as HDM, without a better understanding of the role that allergens play in the complex pathophysiology of AD does not seem propitious at this time.

### Problems of allergen-specific immunotherapy for children with AD

Some final disadvantages to SIT are the low adherence due to time, cost, side effect profile, and difficulty in the route of administration. Immunotherapy has been proven to be a safe treatment method, but it still carries certain risks. SCIT has a higher potential for systemic reactions than SLIT. A 10-year retrospective analysis of general safety of SCIT showed that 5.2 % of patients experienced a systemic allergic reaction in response SCIT injections [5]. Most of the systemic reactions were mild, with another study estimating that a severe systemic reaction occurs between .002 and .0076 % of injections [5]. Despite being small, adverse event rates are not negligible, especially when considering that these therapies are often administered weekly and recommended to last up to 3 years to obtain the ideal effects. SLIT is tolerated better, with fewer adverse events, than SCIT; gastrointestinal side effects are more common than in SCIT. The most common adverse effect of SLIT is a local mucosal reaction, including swelling, pruritus, or dysesthesia, which may occur in up to 75 % of all patients [5]. This type of reaction most commonly occurs during the initiation period of a SLIT regimen and usually subsides within 1 to 3 weeks of starting the treatment. These reactions could promote noncompliance in patients. In one of the few studies investigating the compliance of SLIT in children, a population under 6 years old 46 % of patients discontinued therapy due to a combination of mild, localized adverse events, and the discomfort and/or the difficulty of the route of administration [40]. With SCIT, the patient is required to wait in the providers’ care following dose administration in order to rule out a systemic reaction, which often occur within the first 30 min. This waiting period presents an added burden to the patient but is necessary to ensure overall safety. Although systemic reactions are rarer in SLIT, there is the added risk of delayed treatment since the patient would not be under direct observation of a healthcare provider at the time of the event.

## Conclusion

The summary of the data at present does not warrant the use of SIT in children with AD. While we keep an open mind, problems with selection of the appropriate treatment population, how we handle false positive reactions, and the concerns that polysensitization make it very difficult to interpret what is clinically significant, warrant more studies before SIT can be generally recommended. Also, the potential for the misdirection of time and energy away from skin directed therapy could negatively impact on AD outcomes.

## Abbreviations

AD: Atopic Dermatitis
AR: Allergic Rhinitis
FA: Food Allergy
HDM: House Dust Mite
PPV: Positive Predictive Value
SCIT: Subcutaneous Immunotherapy
SIT: Specific Immunotherapy
SLIT: Sublingual Immunotherapy
SPT: Skin Prick Testing

## References

[1] Bieber T (2010). Atopic Dermatitis. Ann Dermatol.

[2] Hogan MB, Peele K, Wilson NW (2012). Skin Barrier Function and Its Importance at the Start of the Atopic March. J Allergy.

[3] Irvine AD, McLean WH, Leung DY (2011). Filaggrin Mutations Associated with Skin and Allergic Diseases. N Engl J Med.

[4] Suárez-Fariñas M, Dhingra N, Gittler J, Shemer A, Cardinale I, de Guzman SC (2013). Intrinsic Atopic Dermatitis Shows Similar TH2 and Higher TH17 Immune Activation Compared with Extrinsic Atopic Dermatitis. J Allergy Clin Immunol.

[5] Pfaar O, Bachert C, Bufe A, Buhl R, Ebner C, Eng P (2014). Guideline on Allergen-specific Immunotherapy in IgE-mediated Allergic Diseases. Allergo J Int Allergo.

[6] Sicherer SH, Wood RA (2011). Allergy Testing in Childhood: Using Allergen-Specific IgE Tests. Pediatrics.

[7] Pastorello EA, Incorvaia C, Ortolani C, Bonini S, Canonica GW, Romagnani S (1995). Studies on the Relationship between the Level of Specific IgE Antibodies and the Clinical Expression of Allergy: I. Definition of Levels Distinguishing Patients with Symptomatic from Patients with Asymptomatic Allergy to Common Aeroallergens. J Allergy Clin Immunol.

[8] Ricci G, Patrizi A, Baldi E, Menna G, Tabanelli M, Masi M (2006). Long-term Follow-up of Atopic Dermatitis: Retrospective Analysis of Related Risk Factors and Association with Concomitant Allergic Diseases. J Am Acad Dermatol.

[9] Flohr C, Johansson SG, Wahlgren CF, Williams H (2004). How Atopic Is Atopic Dermatitis?. J Allergy Clin Immunol.

[10] Yang H, Xiao YZ, Luo XY, Tan Q, Wang H (2014). Diagnostic Accuracy of Atopy Patch Tests for Food Allergy in Children with Atopic Dermatitis Aged Less than Two Years. Allergol Immunopathol (Madr).

[11] Bergmann MM, Caubet JC, Boguniewicz M, Eigenmann PA (2013). Evaluation of Food Allergy in Patients with Atopic Dermatitis. J Allergy Clin Immunol Pract.

[12] Darsow U, Vieluf D, Ring J (1999). Evaluating the Relevance of Aeroallergen Sensitization in Atopic Eczema with the Atopy Patch Test: A Randomized. Double-blind Multicenter Study. JAAD.

[13] Fatteh S, Rekkerth DJ, Hadley JA (2014). Skin Prick/puncture Testing in North America: A Call for Standards and Consistency. Allergy, Asthma Clin Immunol.

[14] Schäfer T (2008). The impact of allergy on atopic eczema from data from epidemiological studies. Curr Opin Allergy Clin Immunol.

[15] Pajno GB, Caminiti L, Vita D, Barberio G, Salzano G, Lombardo F (2007). Sublingual Immunotherapy in Mite-sensitized Children with Atopic Dermatitis: A Randomized, Double-blind. Placebo-controlled Study. J Allergy Clin Immunol.

[16] Laske N, Niggemann B (2004). Does the Severity of Atopic Dermatitis Correlate with Serum IgE Levels?. Pediatr Allergy Immunol.

[17] Schäfer T, Heinrich J, Wjst M, Adam H, Ring J, Wichmann HE (1999). Association between Severity of Atopic Eczema and Degree of Sensitization to Aeroallergens in Schoolchildren. J Allergy Clin Immunol.

[18] Depner M, Ege MJ, Genuneit J, Pekkanen J, Roponen M, Hirvonen MR (2013). Atopic Sensitization in the First Year of Life. J Allergy Clin Immunol.

[19] De Benedictis FM, Franceschini F, Hill D, Naspitz C, Simons FER, Wahn U (2009). The Allergic Sensitization in Infants with Atopic Eczema from Different Countries. Allergy.

[20] Bath-Hextall F, Delamere FM, Williams HC (2009). Dietary Exclusions for Improving Established Atopic Eczema in Adults and Children: Systematic Review. Allergy.

[21] Sicherer SH (1999). Food Allergy: When and How to Perform Oral Food Challenges. Pediatr Allergy Immunol.

[22] Werfel T, Ballmer-Weber B, Eigenmann PA, Niggemann B, Rancé F, Turjanmaa K (2007). Eczematous Reactions to Food in Atopic Eczema: Position Paper of the EAACI and GA2LEN. Allergy.

[23] Illi S, von Mutius E, Lau S, Nickel R, Grüber C, Niggemann B (2004). The Natural Course of Atopic Dermatitis from Birth to Age 7 Years and the Association with Asthma. J Allergy Clin Immunol.

[24] Du Toit G, Roberts G, Sayre PH, Plaut M, Bahnson HT, Mitchell H (2013). Identifying Infants at High Risk of Peanut Allergy: The Learning Early About Peanut Allergy (LEAP) Screening Study. J Allergy Clin Immunol.

[25] Werfel T, Breuer K (2004). Role of Food Allergy in Atopic Dermatitis. Curr Opin Allergy Clin Immunol.

[26] Sicherer SH, Sampson HA (1999). Food hypersensitivity and atopic dermatitis: pathophysiology, epidemiology, diagnosis, and management. J Allergy Clin Immunol.

[27] Silverberg JI, Simpson EL (2013). Association between severe eczema in children and multiple comorbid conditions and increased healthcare utilization. Pediatr Allergy Immunol.

[28] Burks AW, Mallory SB, Williams LW, Shirrel MA (1988). Atopic dermatitis: Clinical relevance of food hypersensitivity reactions. J Pediatr.

[29] Lee J, Park CO, Lee KH (2015). Specific Immunotherapy in Atopic Dermatitis. Allergy, Asthma Immunol Res.

[30] Jones SM, Burks AW, Dupont C (2014). State of the Art on Food Allergen Immunotherapy: Oral, sublingual, and Epicutaneous. J Allergy Clin Immunol.

[31] Boyce JA, Assa’ad A, Burks AW, Jones SM, Sampson HA, Wood RA (2011). Guidelines for the diagnosis and management of food allergy in the United States: summary of the NIAID-Sponsored Expert Panel report. J Am Acad Dermatol.

[32] Werfel T, Heratizadeh A, Niebuhr M, Kapp A, Roesner LM, Karch A, et al. Exacerbation of Atopic Dermatitis on Grass Pollen Exposure in an Environmental Challenge Chamber. J Allergy Clin Immunol. 2015;136(1). 10.1016/j.jaci.2015.04.015 26044854

[33] Akdis CA, Akdis M, Bieber T, Bindslev-Jensen C, Boguniewicz M, Eigenmann P (2006). Diagnosis and Treatment of Atopic Dermatitis in Children and Adults: European Academy of Allergology and Clinical Immunology/American Academy of Allergy, Asthma and Immunology/PRACTALL Consensus Report. J Allergy Clin Immunol.

[34] Bussmann C, Bockenhoff A, Henke H, Werfel T, Novak N (2006). Does Allergen-specific Immunotherapy Represent a Therapeutic Option for Patients with Atopic Dermatitis?. J Allergy Clin Immunol.

[35] Galli E, Chini L, Nardi S, Benincori N, Panei P, Fraioli G (1994). Use of a specific oral hyposensitization therapy to Dermatophagoides pteronyssinus in children with atopic dermatitis. Allergol Immunopathol (Madr).

[36] Glover MT, Atherton DJ (1992). A Double-blind Controlled Trial of Hyposensitization to Dermatophagoides Pteronyssinus in Children with Atopic Eczema. Clin Exp Allergy.

[37] Warner JO, Price JF, Soothill JF, Hey EN (1978). Controlled Trial Of Hyposensitisation To *Dermatophagoides Pteronyssinus* In Children With Asthma. Lancet.

[38] Gehlhar K, Schlaak M, Becker W, Bufe A (1994). Monitoring Allergen Immunotherapy of Pollen-allergic Patients: The Ratio of Allergen-specific IgG4 to IgG1 Correlates with Clinical Outcome. Clin Exp Allergy.

[39] Sánchez Caraballo JM, Cardona Villa R. Clinical and Immunological Changes of Immunotherapy in Patients with Atopic Dermatitis: Randomized Controlled Trial. ISRN Allergy. 2012:1–9. 10.5402/2012/183983 PMC3658480 23724240

[40] Pajno GB, Caminiti L, Crisafulli G, Barberi S, Landi M, Aversa T (2012). Adherence to sublingual immunotherapy in preschool children. Pediatr Allergy Immunol.

